# Clinical feasibility of a contactless multiparameter continuous monitoring technology for neonates in a large public maternity hospital in Nairobi, Kenya

**DOI:** 10.1038/s41598-022-07189-1

**Published:** 2022-02-23

**Authors:** Amy Sarah Ginsburg, Sahar Zandi Nia, Dorothy Chomba, Dustin Dunsmuir, Mary Waiyego, Jesse Coleman, Roseline Ochieng, Sichen Liu, Guohai Zhou, J. Mark Ansermino, William M. Macharia

**Affiliations:** 1 Clinical Trials Center, University of Washington, Building 29, Suite 250, 6200 NE 74th Street, Seattle, WA 98115 USA; 2 Department of Anesthesiology, The University of British Columbia, Vancouver, BC Canada; 3 Department of Pediatrics, Aga Khan University, Nairobi, Kenya; 4 Pumwani Maternity Hospital, Nairobi, Kenya; 5 Evaluation of Technologies for Neonates in Africa, Seattle, WA USA; 6 Center for Clinical Investigation, Brigham and Women’s Hospital, Boston, MA USA

**Keywords:** Health care, Medical research

## Abstract

Multiparameter continuous physiological monitoring (MCPM) technologies are critical in the clinical management of high-risk neonates; yet, these technologies are frequently unavailable in many African healthcare facilities. We conducted a prospective clinical feasibility study of EarlySense’s novel under-mattress MCPM technology in neonates at Pumwani Maternity Hospital in Nairobi, Kenya. To assess feasibility, we compared the performance of EarlySense’s technology to Masimo’s Rad-97 pulse CO-oximeter with capnography technology for heart rate (HR) and respiratory rate (RR) measurements using up-time, clinical event detection performance, and accuracy. Between September 15 and December 15, 2020, we collected and analyzed 470 hours of EarlySense data from 109 enrolled neonates. EarlySense’s technology’s up-time per neonate was 2.9 (range 0.8, 5.3) hours for HR and 2.1 (range 0.9, 4.0) hours for RR. The difference compared to the reference was a median of 0.6 (range 0.1, 3.1) hours for HR and 0.8 (range 0.1, 2.9) hours for RR. EarlySense’s technology identified high HR and RR events with high sensitivity (HR 81%; RR 83%) and specificity (HR 99%; RR 83%), but was less sensitive for low HR and RR (HR 0%; RR 14%) although maintained specificity (HR 100%; RR 95%). There was a greater number of false negative and false positive RR events than false negative and false positive HR events. The normalized spread of limits of agreement was 9.6% for HR and 28.6% for RR, which met the a priori-identified limit of 30%. EarlySense’s MCPM technology was clinically feasible as demonstrated by high percentage of up-time, strong clinical event detection performance, and agreement of HR and RR measurements compared to the reference technology. Studies in critically ill neonates, assessing barriers and facilitators to adoption, and costing analyses will be key to the technology’s development and potential uptake and scale-up.

## Introduction

With an estimated 6700 newborn deaths every day globally, 47% of all deaths in children under 5 years of age occurred within the first month of life^1^. The highest neonatal mortality rate is in sub-Saharan Africa with 27 deaths per 1000 live births, a rate 10 times higher than rates in high-income countries^2^. In high-income countries, multiparameter continuous physiological monitoring (MCPM) technologies can be critical in the clinical management of high-risk neonates; yet, these technologies are frequently unavailable in resource-constrained settings. At Kenyatta National Hospital in Nairobi, only 3 to 24% of neonates were observed to have their vital signs recorded within the first hour of life, and more than half (56%) did not receive heart rate (HR) or respiratory rate (RR) recordings on their first day of hospital admission^3^. At 6 hospitals in Nairobi county, missed vital signs monitoring and other nursing tasks have been associated with nursing shortages and high patient workloads^4^. MCPM technologies could help to improve quality of neonatal care by expanding nurses’ capacities to monitor more neonates regularly and efficiently. There is a need for innovations in neonatal care that allow for early detection of critical events and timely intervention for major morbidities and are appropriate for use in resource-constrained health facilities^5,6^.

**Figure 1:**
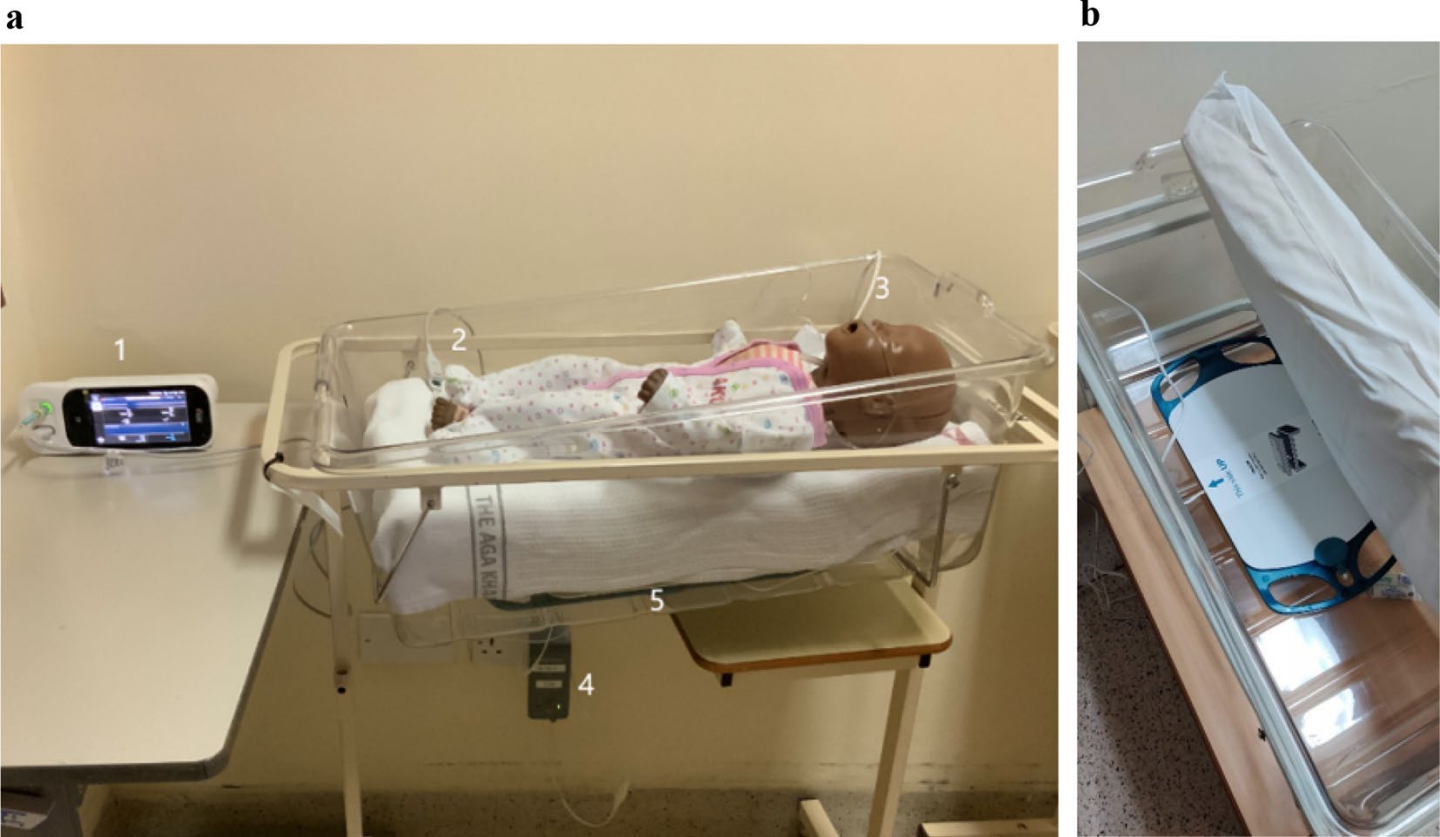
(**a**) Overview of the research set-up showing Masimo's Rad-97 technology with touchscreen interface (1), pulse oximeter probe (2), and NomoLine nasal cannula for capnography (3), and EarlySense’s processing unit (4) and under-mattress sensor (5). (**b**) Close-up of EarlySense’s sensor under a mattress. EarlySense’s sensor is connected to the processing unit that processes, stores data and sends results wirelessly to the remote display unit where the data are presented.

EarlySense’s Insight MCPM technology is a wireless, contactless, piezoelectric sensor pad that can be placed under a neonate’s mattress to detect ballistic vibrations from respiratory chest wall movement and cardioballistic movements from ejection of blood (Fig. 1)^7^. Information from the sensor pad is analyzed using artificial intelligence-based analytics and transmitted to a monitor to provide alert indications and vital sign trends. A report that includes HR, RR, movement readings, and alarms is automatically generated and can be printed and/or integrated into hospital electronic medical record systems. The projected cost of commercial acquisition of EarlySense’s technology is about $150 USD per unit, with no disposable costs greater than the purchase cost over the life of the technology. In a previous clinical trial, we evaluated the accuracy of this novel MCPM technology to measure HR and RR in neonates when compared to a verified reference technology^8^. We also completed qualitative assessments of the feasibility, usability, and acceptability among healthcare personnel and caregivers by conducting in-depth interviews and observations^9,10^.

While medical technologies may be accurate in more controlled settings, also critical to evaluate are their accuracy and the clinical feasibility of use in uncontrolled, real-world settings. These types of evaluation are often not adequately conducted, which has implications for the technology’s eventual adoption, uptake, and scale-up. A novel medical technology’s use in practice may be limited if clinical feasibility performance is not evaluated and the findings incorporated during technology development. To address this, we evaluated EarlySense’s MCPM technology’s clinical feasibility in a large public maternity hospital in Africa. We specifically assessed EarlySense’s technology using objective measures of feasibility that included up-time (periods of adequate signal quality), clinical event detection performance, and sustained accuracy in a real-world environment^11^.

## Methods

### Study design and participants

This prospective, observational, facility-based, clinical validation study was conducted in Nairobi, Kenya at Pumwani Maternity Hospital (PMH), the largest referral maternity hospital in sub-Saharan Africa. PMH has no neonatal intensive care unit. To recruit participants, trained study clinicians approached caregivers of neonates delivered at or admitted to PMH, obtained informed consent, and assessed the neonate for all inclusion and exclusion criteria (Table 1). Final eligibility determination was dependent on the results of the medical history, clinical examination, appropriate understanding of the study by the caregiver, and completion of the written informed consent process. Caregivers who were included in the study gave informed consent for both themselves and their respective neonate to participate.

**Table 1:** Study eligibility criteria, endpoints, and definitions.

**Eligibility criteria**	
Inclusion	Neonate with corrected age of < 28 days requiring admission to the high dependency unit at Pumwani Maternity Hospital for prematurity or other clinical indication(s) based on the attending physician’s assessment Caregiver(s) willing and able to provide informed consent and available for follow-up for the duration of the study
Exclusion	Receiving continuous positive airway pressure or mechanical ventilation Skin abnormalities in the nasopharynx and/or oropharynx Contraindication to skin sensor application Known arrhythmia Congenital abnormality requiring major surgical intervention Any medical or psychosocial condition or circumstance that would interfere with study conduct or for which study participation could put the neonate’s health at risk
**Study endpoints**	
Up-time duration of EarlySense’s technology compared to the reference technology Diagnostic performance of EarlySense’s technology compared to the reference technology for clinical event detection including sensitivity, specificity, positive predictive value, negative predictive value, and ratio of false negative-to-false positive events Agreement between EarlySense’s technology and the reference technology for heart rate (HR) and respiratory rate (RR)	
**Study definitions**	
Total time attached	Measured in minutes as non-zero values recorded by the technology starting 10 min after technology placement and 5 min before disconnection; the 5-min periods before and after neonate removal from the mattress (EarlySense’s technology) or disconnection (reference technology) were also removed
Up-time	Measured in minutes as the total time the sensor was attached that met the a priori-identified *adequate signal quality* limits for each technology
Signal quality, high and adequate	**HR** EarlySense’s technology—*high signal quality* for every second, we evaluated the preceding 59 s in addition to the current second to ensure that at least 30 (50%) seconds demonstrated a proprietary HR signal quality index ≥ 70; *adequate signal quality* for every second, we evaluated the preceding 59 s in addition to the current second to ensure that at least 15 (25%) seconds demonstrated a proprietary HR signal quality index ≥ 25 Reference technology—for every second, we evaluated the preceding 59 s in addition to the current second to ensure that at least 30 (50%) seconds demonstrated a signal quality index (Masimo SQI) > 150 **RR** EarlySense’s technology—*high signal quality* for every second, we evaluated the preceding 59 s in addition to the current second to ensure that at least 42 (70%) seconds demonstrated a proprietary RR signal quality index ≥ 70; *adequate signal quality* for every second, we evaluated the preceding 59 s in addition to the current second to ensure that at least 15 (25%) seconds demonstrated a proprietary RR signal quality index ≥ 25 Reference technology—for every second, we evaluated the preceding 59 s in addition to the current second to ensure that at least 30 (50%) seconds demonstrated no capnography exceptions, indicating low RR quality (RR exceptions ≤ 1), and a capnography quality score ≥ 2
Event second	Any second that contains a high or low HR or RR event for either EarlySense’s technology or the reference technology
Event window	A 10-min window centered from 5 min before to 5 min after the first *event second* noted by the reference technology; no overlapping windows are allowed, so *event seconds* less than 5 min from the end of the previous *event window* result in the new *event window* starting immediately following the previous window
True positive event	Any reference technology *event window* containing at least 1 *event second* identified by EarlySense’s technology
False negative event	A reference technology *event window* containing no *event seconds* recorded by EarlySense’s technology
False positive event	An event recorded by EarlySense’s technology outside the reference technology’s *event window*
True negative event	Any 10-min window with no events recorded by either EarlySense’s technology or the reference technology
Clinically significant event	Any *false negative* or *false positive event* that would likely require a clinician to institute a change in clinical practice

The study was conducted in accordance with the Declaration of Helsinki and Guideline for Good Clinical Practice/International Standards Organization (ISO) 14155 to ensure accurate, reliable, and consistent data collection. The study protocol was approved by Western Institutional Review Board (20191102), Aga Khan University Nairobi Research Ethics Committee (2019/REC-02), and Kenya Pharmacy and Poisons Board (19/05/02/2019(078)). The trial was registered with ClinicalTrials.gov, NCT03920761.

### Procedures

Each enrolled neonate with one neonate per cot was simultaneously and continuously monitored with EarlySense’s technology and Masimo’s Rad-97 pulse CO-oximeter with capnography as the verified reference standard. We selected this reference technology based on its ability to extract and record high resolution data, and perform neonatal capnography and pulse oximetry, and its compact design enabling bedside monitoring. Real-time HR and RR data were collected from both technologies for a duration of at least 1 hour and continued until neonates were discharged and exited from the study. Up-time, clinical event detection performance, and HR and RR measurements data were collected by both technologies (Table 1). Great care was taken to ensure participation in the study did not interfere with or unnecessarily delay the clinical care of the neonates.

### Outcomes

Study outcomes included comparisons between EarlySense’s technology’s and the reference technology’s total time attached and up-time, event detection of high and low HR and RR events, and agreement of HR and RR measurements (Table 1).

### Data processing and analysis

We calculated the total number of minutes the sensors were attached and the up-time for each technology. The first step in the automated event detection process was to assess the quality of the measurements from both technologies, with periods of adequate signal quality data referred to as up-time (Table 1). For EarlySense’s technology, we obtained HR and RR every second along with a proprietary signal quality index. We retrieved raw data collected in real-time from the reference technology with a custom Android application. Data was parsed in C (Dennis Ritchie & Bell Labs, USA) to obtain plethysmograph waveform and plethysmograph quality index (PO-SQI) data at 62.5 Hz (Hz), and capnography (carbon dioxide (CO_2_)) waveform data at approximately 20 Hz. We analyzed CO_2_ waveform data using a breath detection algorithm developed in MATLAB (Math Works, USA) based on adaptive pulse segmentation^12^. HR and RR were obtained from intra-beat and intra-breath intervals, respectively. We developed a custom algorithm based on capnography features to determine the capnography quality index (CO_2_-SQI). For detecting events, upper limits for HR and RR were individualized for each neonate and were calculated to be 20% for HR and 15% for RR greater than the respective baseline value once the neonate was settled (approximately 15 minutes after monitoring was started), but no less than 140 beats/minute for HR and 40 breaths/minute for RR. Lower limits for HR and RR for all neonates were static values of 80 beats/minute for HR and 15 breaths/minute for RR. Using EarlySense’s technology, high and low HR and RR events were identified in real-time using the above a priori-determined criteria; no modification or post-processing was performed. Reference technology data were processed following data collection to extract HR and RR values and identify high and low events. We developed a custom algorithm to identify, aggregate, and categorize all high and low HR and RR events.

**Figure 2:**
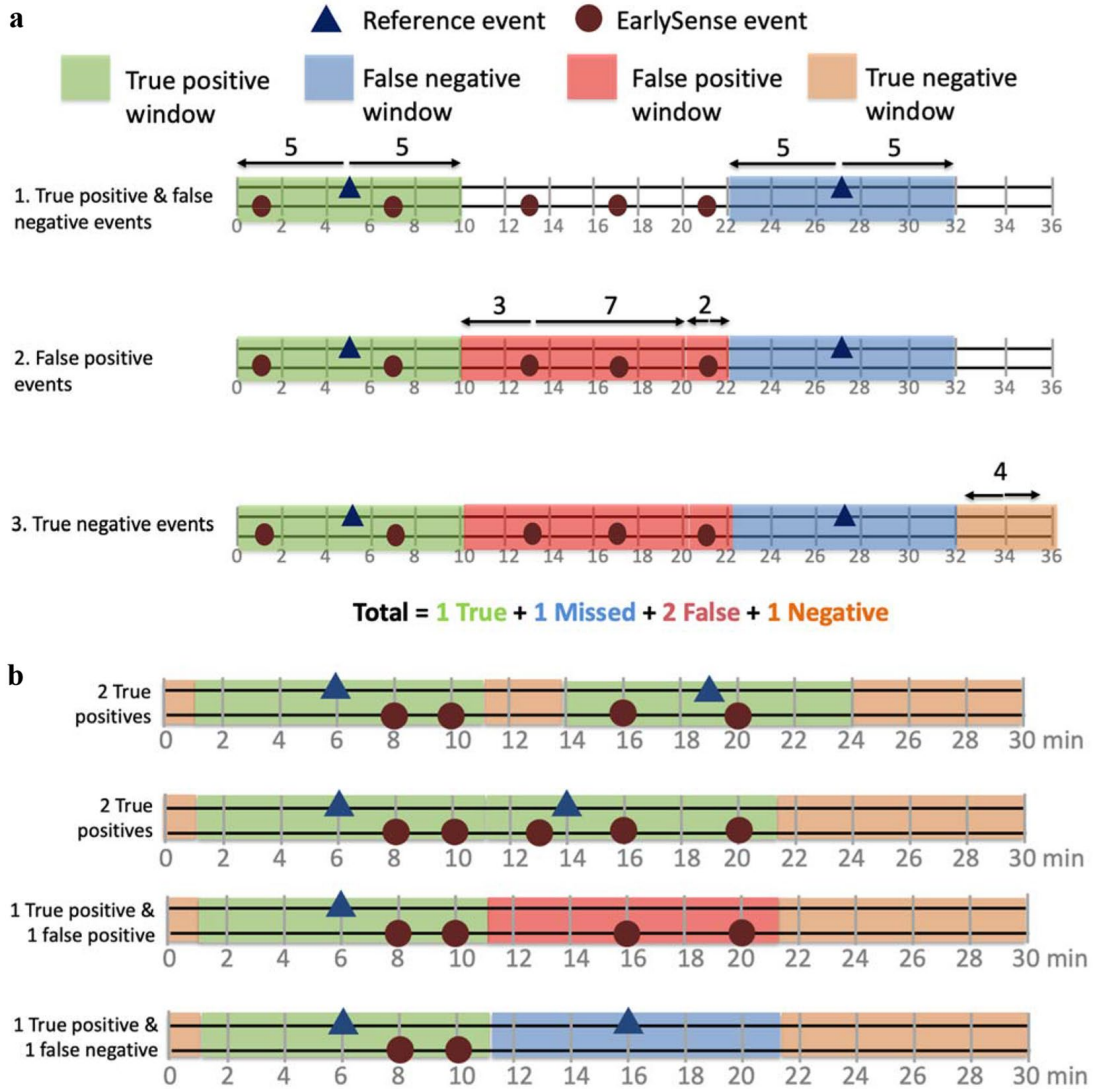
Event identification schema by automated algorithm. (**a**) Events were identified in order: true positive and false negative, false positive, and true negative events. (**b**) Examples of how the algorithm identified events in different scenarios.

High and low HR and RR events were identified during overlapping up-time from both technologies. Events were identified by looking at the previous minute of data for both technologies. An event was identified if the 1-minute median and the recent 10-second median both met adequate signal quality and the upper or lower alarm values for HR or RR. We used a custom algorithm to aggregate events from the two technologies into 10-minute windows categorized as true positive, false negative, false positive, or true negative for both high and low HR and RR individually (Table 1, Fig. 2). To gather insight around the signal quality threshold for EarlySense’s technology, we conducted two analyses, one including only the a priori higher signal quality (≥ 70) data and one including all adequate signal quality (≥ 25) data.

Manual adjudication of algorithmically identified events detected in the higher signal quality data was performed by a panel of trained neonatologists to review false negative and false positive events and to identify those that were clinically significant (Table 1). Adjudication packages consisting of visualizations of false negative and false positive HR and RR events and adjudication checklists (Supplementary Fig. S1) were developed and disseminated to the panel of adjudicators. To ensure the robustness of this process, false negative and false positive events were independently reviewed by two adjudicators and a third adjudicator reconciled the results if there was disagreement. Confusion matrices were generated to evaluate the EarlySense technology’s event detection ability. The matrices categorized all events for both pre- and post-adjudication results and included accuracy, sensitivity, specificity, positive predictive value (PPV), negative predictive value (NPV), and false negative-to-false positive ratio for high and low HR and RR.

Using the higher signal quality dataset, agreement between EarlySense’s technology and the reference technology was evaluated as the normalized bias and the normalized spread between the 95% limits of agreement (LOA) calculated by dividing the bias and spread between the 95% LOA by the overall reference HR or RR mean value^13^. Based on the reference technology verification phase, an acceptable a priori-identified normalized spread between the 95% upper and lower LOA of 30% was selected for both RR and HR^14^. We also calculated the root-mean-square deviation (RMSD) for each comparison. Data analyses were completed with R version 4.0.2.

## Results

Between September 15 and December 15, 2020, 116 neonates were enrolled, 109 of whom were included for analysis (Supplementary Fig. S2). Seven neonates were excluded due to less than 1 hour recording duration with EarlySense’s technology. No adverse events occurred due to monitoring with the technologies and after monitoring was finished, all enrolled neonates remained in stable condition until discharge to home. Included in the analysis were 57 females and 52 males. Median estimated age was 0 (interquartile range 0, 2) days, median gestational age was 39 (range 37, 41) weeks, and median weight was 3.0 (range 2.3, 3.4) kilograms. Common primary and secondary diagnoses included sepsis or suspected sepsis (26; 23.9%), asphyxia (25; 22.9%), respiratory distress syndrome (24; 22.0%), prematurity (17; 15.6%), meconium aspiration syndrome (16; 14.7%), and jaundice (16; 14.7%) (Supplementary Table S3).

**Figure 3:**
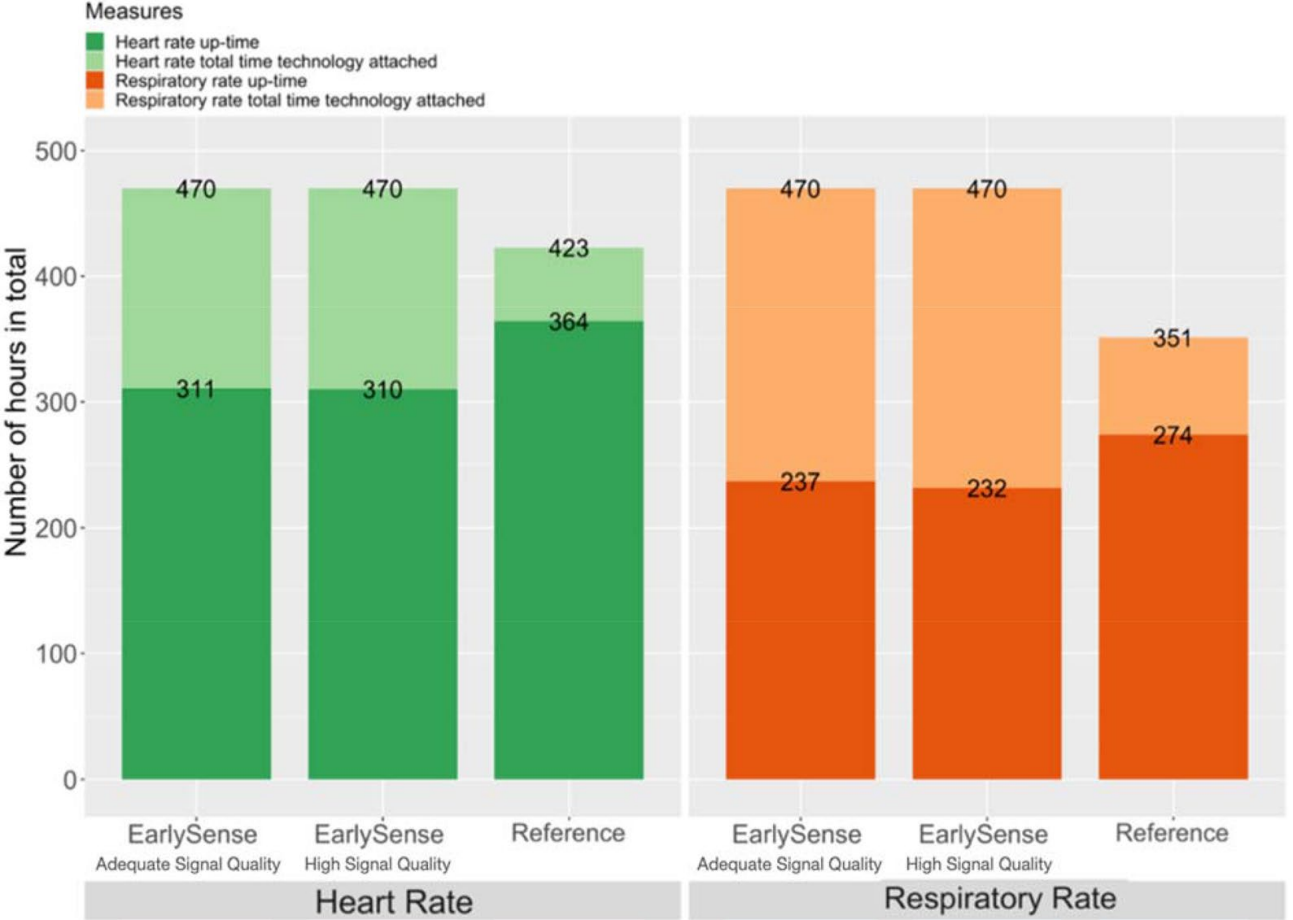
Total time technology attached and up-time.

We collected and included 470 hours of data for HR and RR from EarlySense’s technology in the higher signal quality dataset, and 423 hours of HR data and 351 hours of RR data from the reference technology (Fig. 3). In the higher signal quality dataset analysis, the difference compared to the reference was a median of 0.6 (range 0.1, 3.1) hours for HR and 0.8 (range 0.1, 2.9) hours for RR; overall, this was 54 (16%) hours less for HR and 42 (17%) hours less for RR. In the higher signal quality dataset analysis, the mean up-time per neonate for EarlySense’s technology was 2.9 (range 0.8, 5.3) hours for HR and 2.1 (range 0.9, 4.0) hours for RR; the mean up-time per neonate for the reference technology was 3.3 (range 1.2, 5.7) hours for HR and 2.5 (range 0, 4.4) hours for RR. Reducing the quality threshold to adequate (≥ 25) resulted in one additional hour of HR and 5 additional hours of RR of total up-time for EarlySense’s technology.

EarlySense’s technology identified the majority (67%) of HR events (Table 2). There was a greater number of false negative and false positive RR events than false negative and false positive HR events. Using the higher signal quality dataset, EarlySense’s technology's high HR and RR event detection showed high accuracy, sensitivity, specificity, PPV, and NPV, and a low false negative-to-false positive events ratio (Supplementary Fig. S4). EarlySense’s technology's low HR and RR event detection demonstrated high accuracy, specificity, NPV, and false negative-to-false positive events ratio, but were lower in sensitivity and PPV. The few additional hours of adequate signal quality data in our analyses resulted in minimal overall changes in event detection.

**Table 2:** Clinical event detection.

	High heart rate			Low heart rate			High respiratory rate			Low respiratory rate		
	Pre-A	Pre-H	Post	Pre-A	Pre-H	Post	Pre-A	Pre-H	Post	Pre-A	Pre-H	Post
**Event**												
True positive	65	60	72	0	0	0	634	407	506	10	1	1
True negative	1458	1206	1206	1529	1251	1251	292	198	198	653	279	293
False positive	26	15	10	3	3	3	29	42	12	90	14	1
False negative	20	14	7	1	1	1	411	83	14	12	6	5
**Statistical summary**												
Accuracy	97%	98%	99%	100%	100%	100%	68%	83%	96%	87%	93%	98%
Sensitivity	77%	81%	90%	0%	0%	0%	61%	83%	97%	46%	14%	17%
Specificity	98%	99%	99%	100%	100%	100%	91%	83%	94%	88%	95%	100%
PPV	71%	80%	86%	0%	0%	0%	96%	91%	97%	10%	7%	50%
NPV	99%	99%	99%	100%	100%	100%	42%	71%	93%	98%	98%	98%
FN:FP	1:1.3	1:1.1	1:1.4	1:3	1:3	1:3	1: 0.1	1:0.5	1:0.9	1:7.5	1:2.3	1:0.2

Pre-adjudication with adequate signal quality analysis (Pre-A); pre-adjudication with higher signal quality analysis (Pre-H); post-adjudication (Post); positive predictive value (PPV); negative predictive value (NPV); false negative event to false positive event ratio (FN:FP).

Accuracy = (True positive + True negative)/(True positive + True negative + False negative + False positive); Sensitivity = True positive/(True positive + False negative); Specificity = True negative/(True negative + False positive); PPV = True positive/(True positive + False positive); NPV = True negative/(True negative + False negative).

For HR monitoring, there were minimal changes in accuracy, sensitivity, specificity, PPV, NPV, and false negative-to-false positive events ratio observed between pre- and post-adjudication of events (Table 2, Supplementary Fig. S4). Following adjudication of 178 false negative and false positive events, only 1 false negative high RR event was deemed clinically significant. Performance generally improved following adjudication for RR events; however, there was minimal improvement in low RR event sensitivity (14% to 17%) due to the infrequent occurrence of low RR events.

**Figure 4:**
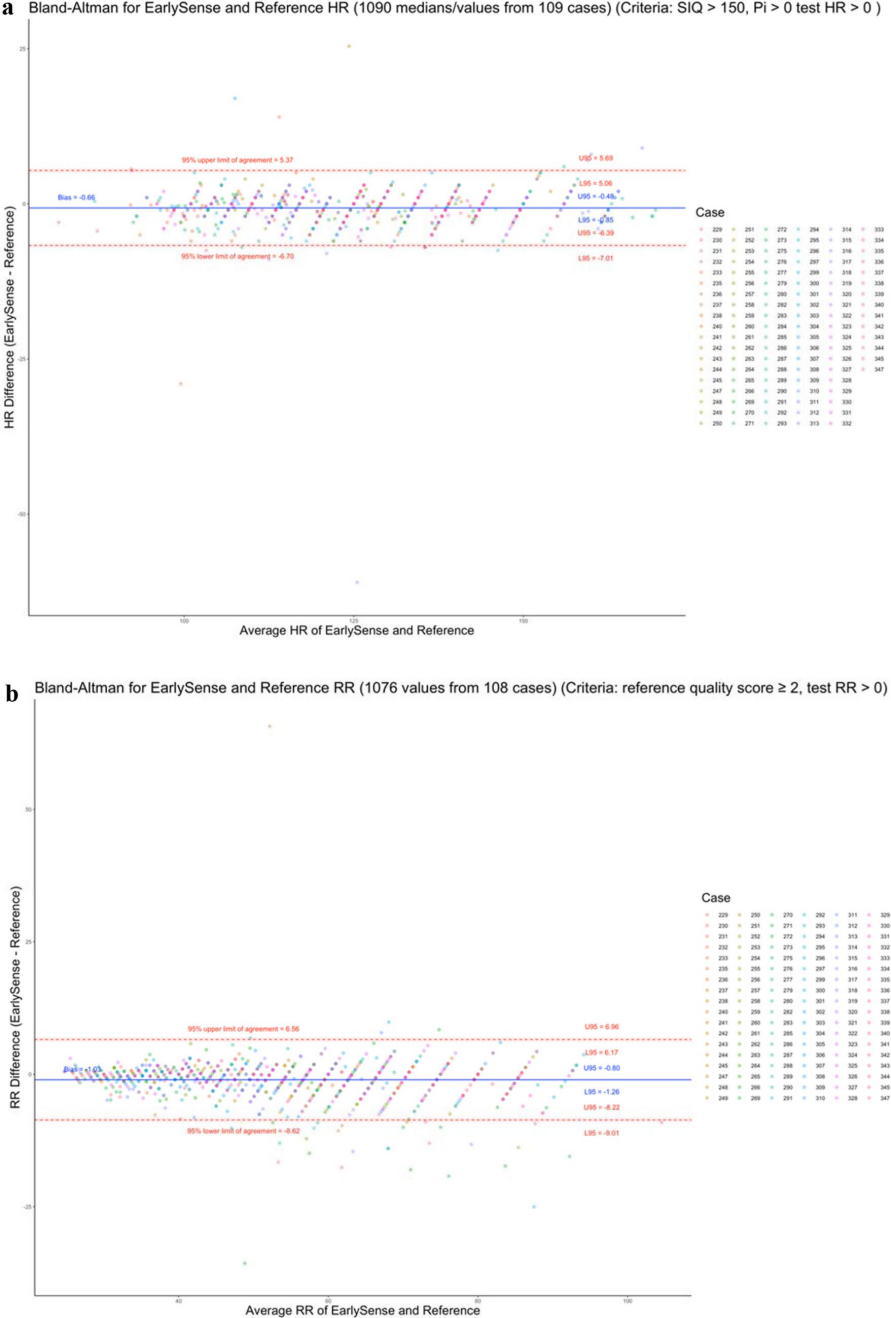
Bland–Altman plots of measured (**a**) heart rate (HR) and (**b**) respiratory rate (RR) as measured by EarlySense’s and the reference technologies. Colors indicate which participant neonate is associated with the measurement pair.

HR agreement indicated a minimal normalized bias of − 0.5% (95% CI − 0.7, − 0.4), a normalized spread of LOA of 9.6%, and a normalized RMSD of 2.5% (Fig. 4a). RR agreement indicated a normalized bias of − 2.0% (95% CI − 2.4, − 1.5), a normalized spread of LOA of 28.6%, and a normalized RMSD of 7.6% (Fig. 4b). The a priori-identified limit of 30% for the normalized spread of LOA was met for both HR and RR.

## Discussion

We found EarlySense’s MCPM technology to be clinically feasible in a large public maternity hospital in Africa, as demonstrated by the technology’s up-time when the neonate was physically on the mattress, the clinical event detection performance, and the agreement of HR and RR measurements compared to the reference technology. Agreement of neonatal HR and RR measurements between EarlySense’s technology and the verified reference technology in this real-world clinical environment was similar to what we found in a more controlled clinical trial at a better-resourced hospital with a neonatal intensive care unit^8^. There was high agreement between EarlySense’s and the reference technologies’ event detection of high and low HR and high RR events, which further improved post-adjudication. With so few low RR events among the neonates we evaluated, we were not able to robustly assess EarlySense’s technology in this respect. Of note, given that EarlySense’s algorithm is programmed to identify apneic events using more than just low RR, their technology does not use low RR as an independent event detection threshold.

To be clinically feasible and effective, a technology would need to avoid excessive alarms resulting from false positive events and suppress transient artifacts without missing clinically significant events. Typically, this would be accomplished by employing an appropriate delay before alarm generation. EarlySense’s technology detected few false positive events. This bodes well for the technology’s ability to reliably detect true positive clinical events and to generate critical alarms to allow healthcare providers to respond without also causing unnecessary alarms that could lead to alarm fatigue.

The major limitation to EarlySense’s under-mattress technology is that continuous monitoring cannot occur when the neonate is out of bed, especially during breastfeeding and kangaroo mother care. Notably, the reference technology’s capnography cannula is also removed when the neonate is out of bed. We did not test EarlySense’s technology when there was more than one neonate per cot although no special mattress or cot was used to improve the performance of the technology; thus, we do not know how EarlySense’s technology would perform when neonates have to share cots.

Our evaluation, analyses, and adjudication may have been constrained by the a priori signal quality limits we chose, which were relatively arbitrary. The a priori signal quality limits were selected to mitigate confusion that could have arisen if poor quality signals were used for event detection. We chose to treat event detection as if it had happened in real-time. Given that only 1 hour of HR and 5 hours of RR data were excluded from the higher signal quality data analysis, these limits may have been appropriate to include the majority of the monitoring data. The analyses including all adequate signal quality data demonstrated marginal degradation in event detection performance for both HR and RR.

There were relatively few clinically significant events, despite the prolonged duration of monitoring, and we did not allow either technology to generate alarms that may have impacted clinical outcomes. Of note, we limited our comparison to the detection of low RR and did not evaluate the detection of apnea since the reference device did not detect any episodes of apnea. A clinically useful monitor should be able to detect life-threatening apnea associated with bradycardia and desaturation. For this reason, direct comparison between EarlySense’s technology and the reference technology was not feasible. Another limitation was that the study population was relatively healthy and critically ill neonates did not appear to be included, and thus, few life-threatening events were recorded. This limits the generalizability of our evaluation; the technology would benefit from being evaluated in different populations and settings. In addition, we did not explore clinical outcomes or impact.

Despite these limitations, the study results from this and a previous accuracy evaluation in Kenya indicate that EarlySense’s contactless MCPM technology performs well when used to measure neonatal HR and RR as compared to the more invasive Masimo Rad-97 reference technology^8^. Evaluating the accuracy and the clinical feasibility of medical technologies remains a critical and necessary step in product development and use. Understanding the technology and its use cases is important to better appreciate how these technologies (and their alarms) could change clinical practice and management of neonates, especially in these resource-constrained settings where the high patient-to-provider ratios severely limits quality of care. Studies of monitoring technologies in neonates have largely been limited to high-income countries. Established methods for continuous HR and RR monitoring exist, but factors such as invasiveness, time-consuming application, and high cost have contributed to feasibility concerns in resource-constrained settings. Novel contactless technologies such as EarlySense’s technology may be able to address these concerns while also avoiding skin irritation and distress to the vulnerable neonate; however, notably, EarlySense’s technology does not include oxygen saturation monitoring which may be considered critical to neonatal care.

Use of clinically accurate and feasible MCPM technologies have the potential to improve quality of neonatal care. It will be critical to evaluate EarlySense’s technology in more preterm and critically ill neonates, assessing the threshold and adaptive alerts provided by the technology, to consider barriers and facilitators to adoption, and to conduct costing studies. This type of information and feedback will be key to the technology’s development as well as its successful uptake and scale-up.

## Supplementary Information

Supplementary Information.

## Data Availability

De-identified individual participant data, raw reference data, and a data dictionary will be made available on publication through moderated approvals supported by Vivli.org. In addition, the study protocol, statistical analysis plan, and the informed consent form will be made available. We have ethics approval to deposit the data in an open access repository upon completion of the study.
