# Supplementary material for: Clinical feasibility of a contactless multiparameter continuous monitoring technology for neonates in a large public maternity hospital in Nairobi, Kenya
Source: Sci Rep. 2022 Feb 23;12:3097. doi: 10.1038/s41598-022-07189-1 (PMC8866488; doi:10.1038/s41598-022-07189-1)
Supplement: Supplementary file 1 — Supplementary Information. [file 41598_2022_7189_MOESM1_ESM.docx]

**Supplementary Tables and Figures**


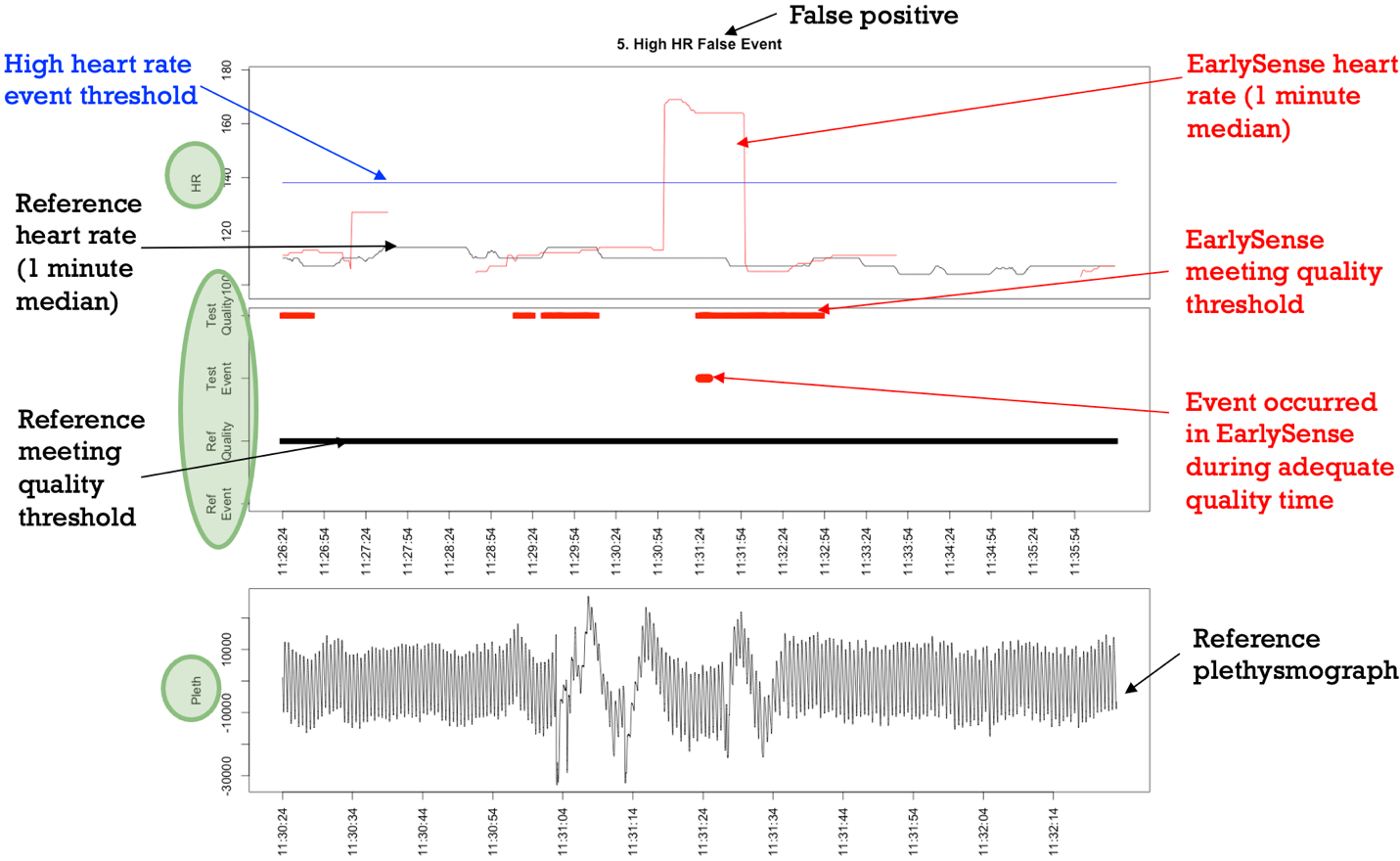


**
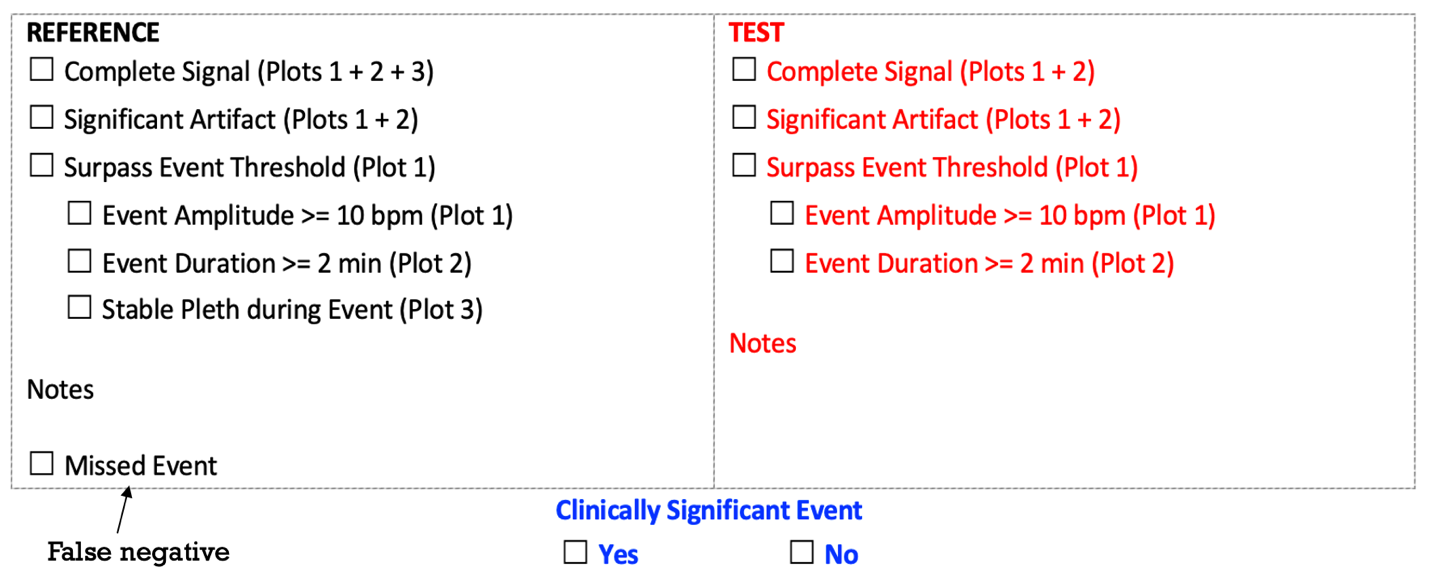
**

**Supplementary Figure S1.** Adjudication form for heart rate. Respiratory rate form resembles heart rate except capnography replaces the plethysmograph.

Neonates assessed for eligibility

(n=123)

Neonates excluded (n=7)

♦  Not meeting inclusion criteria (n= 1)

♦  Declined to participate (n= 4)

♦  Other reasons (n= 2)

Neonates enrolled (n=116)

Neonates’ data excluded (n=7)

♦  Insufficient data recording length (n= 7)

Neonates’ data included in analysis (n=109)

**Supplementary Figure S2.** Flow diagram showing enrolled participants.

| **Primary diagnosis** | **Number** | **Percent** |
| --- | --- | --- |
| Sepsis/suspected sepsis | 23 | 21.1 |
| Asphyxia | 21 | 19.3 |
| Respiratory distress syndrome | 16 | 14.7 |
| Prematurity | 14 | 12.8 |
| Meconium aspiration syndrome | 11 | 10.1 |
| Jaundice | 5 | 4.6 |
| Low birthweight | 5 | 4.6 |
| Macrosomia | 4 | 3.7 |
| Mother VDRL positive | 3 | 2.8 |
| Dehydration | 2 | 1.8 |
| Hypoxia | 2 | 1.8 |
| Hepatitis B exposed | 1 | 1.0 |
| Poor latching | 1 | 1.0 |
| None | 1 | 1.0 |
| Total | 109 | 100 |
| **Secondary diagnosis** |  |  |
| Jaundice | 11 | 25.0 |
| Respiratory distress syndrome | 8 | 18.2 |
| Meconium aspiration syndrome | 5 | 11.4 |
| Asphyxia | 4 | 9.1 |
| Low birthweight | 3 | 6.8 |
| Prematurity | 3 | 6.8 |
| Sepsis/suspected sepsis | 3 | 6.8 |
| Convulsions | 1 | 2.3 |
| Dehydration | 1 | 2.3 |
| Hypoglycemia | 1 | 2.3 |
| Macrosomia | 1 | 2.3 |
| Ophthalmia neonatorum | 1 | 2.3 |
| Periorbital sepsis | 1 | 2.3 |
| Vomiting | 1 | 2.3 |
| Total | 44 | 100 |

**Supplementary Table S3.** Primary and secondary diagnoses during enrolled neonates’ current hospitalizations (per hospital medical chart).

**
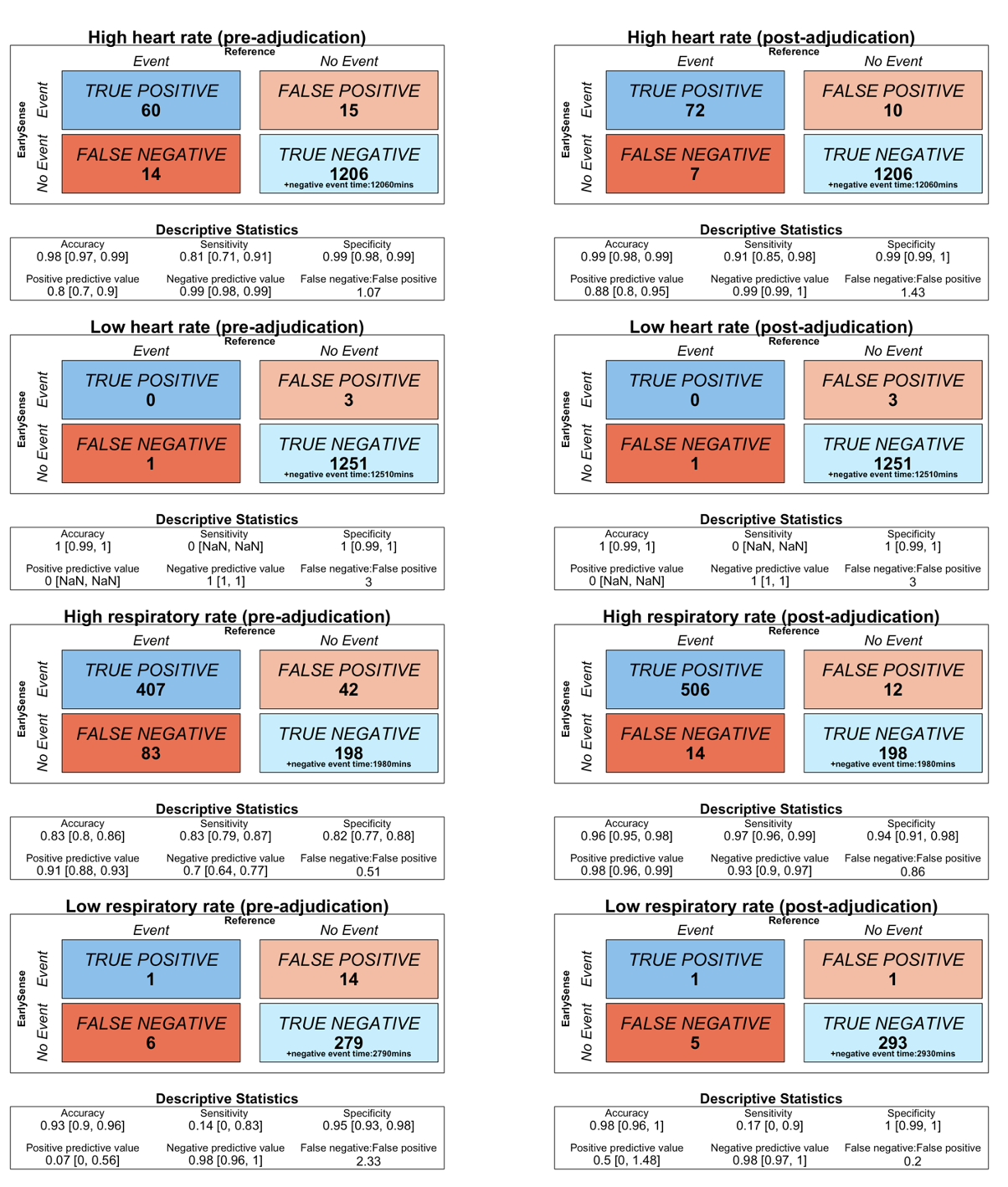
**

**Supplementary Figure S4.** Confusion matrices of high and low heart rate (HR) and respiratory rate (RR) events pre- and post-adjudication for the higher signal quality analysis. 95% confidence intervals in square brackets.
